# Prescribed Performance Bounded-H_∞_ Control for Flexible-Joint Manipulators Without Initial Condition Restriction

**DOI:** 10.3390/s25072195

**Published:** 2025-03-30

**Authors:** Ye Zhang, Ruibo Sun, Jie Shang

**Affiliations:** 1 Sinopec Yangzi Petrochemical Co., Ltd., Nanjing 210048, China; zhangye.yzsh@sinopec.com; 2 School of Electrons and Information Engineering, University of Science and Technology Liaonin, Anshan 114051, China; ruibosun@foxmail.com; 3 CAS Key Laboratory of Magnetic Materials and Devices, Ningbo Institute of Materials Technology and Engineering, Chinese Academy of Sciences, Ningbo 315201, China; 4 Zhejiang Province Key Laboratory of Magnetic Materials and Application Technology, Ningbo Institute of Materials Technology and Engineering, Chinese Academy of Sciences, Ningbo 315201, China

**Keywords:** prescribed performance control, bounded-H_∞_ control, sensor faults, fault-tolerant control

## Abstract

Flexible-joint manipulators have a lightweight nature, compact structure, and high flexibility, making them widely applicable in industrial manufacturing, biomedical instruments, and aerospace fields. However, the inherent flexibility of single-link flexible-joint manipulators (SLFJMs) poses substantial control challenges. Compared to traditional control algorithms, prescribed performance control (PPC) algorithms provide superior transient response and steady-state performance by defining a prescribed performance function. However, existing PPC algorithms are limited to a specific range of system initial states, which reduces the joint manipulator’s operational workspace and weakens the robustness of the control algorithm. To address this issue, this study proposes a prescribed performance bounded-H_∞_ fault-tolerant controller for SLFJMs. By designing an improved tangent-type barrier Lyapunov function (BLF), a prescribed performance controller that is independent of the initial state of the SLFJM is developed. An input control function (ICF) is employed to mitigate the impulse response of the control input, ensuring a smooth transition from zero. Furthermore, the improved tangent-type BLF enables the tracking error to rapidly converge to a small neighborhood of zero. Finally, a stabilization control simulation experiment is conducted; the results validate the effectiveness of the proposed prescribed performance bounded-H_∞_ controller.

## 1. Introduction

Flexible-joint manipulators are widely applied in industrial manufacturing, biomedical instruments, and aerospace fields [1,2,3,4] owing to their lightweight nature, compact structure, and high flexibility. However, strong coupling and nonlinearity due to their inherent flexible characteristics make flexible-joint manipulators prone to residual vibration and large control errors, as well as even cause system instability.

Extensive efforts have been made in the study of single-link flexible-joint manipulator (SLFJM) control for short adjustment time and high motion precision [5,6,7]. In [8], a nonlinear self-tuning PID controller was proposed to control the joint position and link deflection of a flexible joint manipulator while it was subjected to carry different payloads. However, the nonlinear characteristics of SLFJM systems considerably degrade the performance of PID controllers, resulting in a long settling time [9]. To reduce the settling time, sliding mode control is applied to flexible-joint manipulators [10,11]. An extended-state-observer-based adaptive controller, which eliminates joint stiffness uncertainties, has been proposed for SLFJMs in [12]. A singular perturbation theory based decoupling controller was proposed in [13] to solve the problems concerning finite time trajectory tracking. However, chattering in the input control signals, caused by the high-frequency switching of the control input, may lead to unwanted wear and, in some cases, system instability. In addition, neither a PID controller nor a sliding mode controller can explicitly determine the stability time of SLFJM systems.

**Figure 1:**
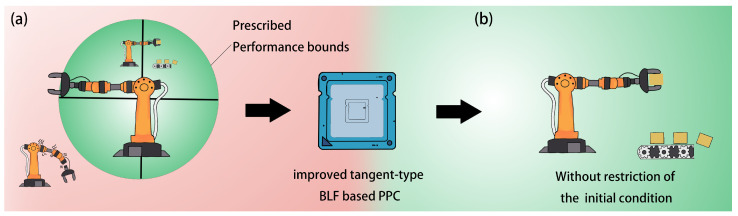
Schematic diagram of the application scenario for a SLMFJ controlled using the improved tangent-type BLF-based PPC method: (**a**) Illustration of the issues with the manipulator controlled by the existing PPC scheme. (**b**) Illustration of the manipulator controlled by the improved tangent-type BLF-based PPC.

To achieve superior transient response and steady-state performance, prescribed performance control (PPC) has been proposed by Bechlioulis in [14]. To reduce tracking errors, the PPC method and the barrier Lyapunov function (BLF) are widely used in SLFJMs. BLF-based methods can be classified into three main types: the tangent-type BLF method [15,16], the integral-type BLF method [17], and the logarithm-type BLF method [18,19]. A tangent barrier-Lyapunov-function-based adaptive event-triggered control for uncertain flexible beam systems was carried out in [20] However, in traditional PPC based on the BLF, there exists an inherent restriction at the initial time, which requires the constrained variable to remain within a predefined region, as depicted in Figure 1a. This limitation arises from the mathematical formulation of the BLF. Consequently, existing PPC algorithms can only be applied within a specific range of the system’s initial state. If the initial condition is completely unknown or the initial constrained variable falls outside the predefined region, achieving the desired control result becomes impossible. To address this issue, this paper presents an improved tangent-type BLF-based PPC, which enables the SLMFJ to be free from the restriction of prescribed performance bounds and can commence operation from any point, as shown in Figure 1b.

Aside from dynamic performance, the fault-tolerant capability of SLFJM systems should also be considered [21,22,23,24]. One common approach is to treat sensor faults as system uncertainties and design a robust controller to mitigate their influence on the associated system [25]. System uncertainties were modeled using a fault loss function in [26], where a finite-time controller was designed to ensure the global exponential stability of an SLFJM system. Some distributed fault-tolerant control algorithms were obtained in [27]. In this study, a similar approach is adopted to address sensor faults. However, conventional control schemes may result in an excessively large step control input at the initial time. An input control function (ICF) is applied to prevent this and ensure that the control signal starts from zero.

The contributions of this paper are summarized as follows:

1. A prescribed finite-time bounded-H_∞_ robust fault-tolerant controller without initial condition restrictions is designed. Unlike the conventional PPC method in [15,16], the controller in this study ensures finite-time stability even when the initial system states violate their constraints.
2. A new control method is applied to an SLFJM. Unlike general control design schemes [18,28], the initial value of the proposed control input is zero. This design prevents the large step impact on the SLFJM.

The remainder of this paper is organized as follows.

Section 2 presents the system description and preliminaries. Section 3 discusses the controller design and stability analysis. Section 4 presents a stabilization control simulation. Section 5 presents the conclusions of this study.

## 2. Problem Formulation and Preliminaries

### 2.1. Plant Formulation

Consider the SLFJM as

(1) $$\begin{array}{c} \left\{\begin{array}{c} I\ddot{q}+K\left(q-\theta \right)+MgL\sin q+d_{1}=0\\ J\ddot{\theta }-K\left(q-\theta \right)+d_{2}=\tau \end{array}\right. \end{array}$$

where *q* and θ denote the angular positions of the link and motor shaft, respectively. τ represents the input control torque of the system. *I* and *J* represent the inertias of the link and motor. *M* denotes the link mass, g represents the gravitational acceleration, *K* represents the spring constant of the joint stiffness, and *L* represents the length of the moment arm. $d_{1}$ and $d_{2}$ denote the unknown disturbances on the motor side and output side, respectively. In this study, *q* and θ were measured using angle sensors.

**Figure 2:**
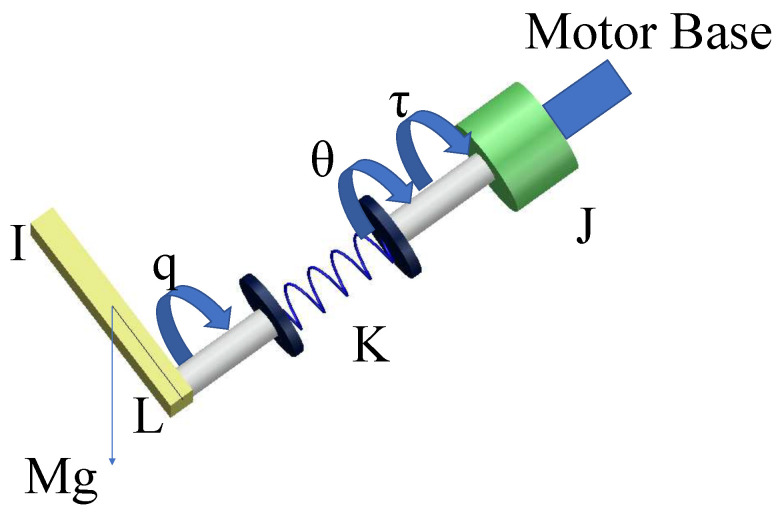
Diagram of the SLFJM.

For clarity, the diagram of the SLFJM is shown in Figure 2.

Define $x_{1}=q,x_{2}=\dot{q},x_{3}=\theta ,x_{4}=\dot{\theta },u=\tau$. Then, the SLFJM can be expressed as

(2) $$\begin{array}{c} \left\{\begin{array}{c} {\dot{x}}_{1}=x_{2}\\ {\dot{x}}_{2}=g_{2}x_{3}+f_{2}\left({\bar{x}}_{2}\right)+d_{2}\\ {\dot{x}}_{3}=x_{4}\\ {\dot{x}}_{4}=g_{4}u+f_{4}\left({\bar{x}}_{4}\right)+d_{4}\\ y=x_{1} \end{array}\right. \end{array}$$

where $f_{2}=-\frac{MgL}{I}\sin x_{1}-\frac{K}{I}x_{1}$, $g_{2}=\frac{K}{I}$, and $f_{4}=\frac{K}{J}\left(x_{1}-x_{3}\right)$, $g_{4}=\frac{1}{J}$.

Moreover, $x={\left[x_{1},x_{2},x_{3},x_{4}\right]}^{T}\in R^{4}$ represents the system state, and *y* represents the system output.

### 2.2. Preliminaries

**Definition** **1** (fault loss function [21])**.** *The sensor output of the ith system $X_{i}$, which is input into the controller, can be modeled as*

(3) $$X_{i}\left(t\right)=o_{i}\left(t\right)x_{i}\left(t\right)$$

*where $o_{i}\left(t\right)$ is the fault loss function, $o_{i}\left(t\right)\in \left(0,1\right)$ and $o_{i}\left(t\right)$ is differentiable.*

In the SLMFJ system proposed in this paper, the sensors operate continuously. Therefore, sensor performance degradation occurs throughout both the transient and steady-state processes of the system.

**Assumption** **1** ([24])**.** *The function $o_{i}\left(t\right)$ satisfies $0\leq \underline{o}\leq o_{i}\left(t\right)<1$, where $\underline{o}$ represents the minimum of $o_{i}\left(t\right)$, and the value of $\underline{o}$ is in the range of 0 to 1.*

**Assumption** **2** ([29])**.** *According to the state-space Equation (2), we can assume that $g_{i}>0$. It is further assumed that there exists an unknown constant $b_{m}$ satisfying $0<b_{m}\leq g_{i}$. Moreover, there exists an unknown constant b such that*

(4) $$b\leq b_{m}\underline{o}\leq o_{i}g_{i}\leq \frac{o_{i}}{o_{i+1}}g_{i}$$

**Lemma** **1** ([30])**.** *Consider a smooth function η(t) satisfying*

(5) $$\begin{array}{c} \left\{\begin{array}{c} \eta (0)=0\\ 0<\eta \left(t\right)<1,0<t<T_{b}\\ \eta \left(t\right)=1,t\geq T_{b} \end{array}\right. \end{array}$$

*where $T_{b}$ is a designed parameter.*

*Let V(t) be a continuous function satisfying $\forall V\left(t\right)\in R^{+}$, and V(0) is bounded. If (6) holds, then*

(6) $$\dot{V}\leq -p\eta V+q$$

*where p>0 and q are constants, so then V is bounded.*

In addition, designate η(t) as an ICF.

**Definition** **2** ([31])**.** *For the system (2), if there is a Lyapunov function V(t), this entails the following:*

*1.*  *V(x(0))>0.*
*2.*  *$V\left(x\left(0\right)\right)+\gamma^{2}\int_{0}^{t}{∥d\left(s\right)∥}^{2}ds\geq \int_{0}^{t}{∥z\left(s\right)∥}^{2}ds$.*

*Therein, z is the tracking error, $d\left(t\right)\in L_{2}\left[0,T\right]$ is the bounded external disturbance, γ denotes a disturbance attenuation coefficient, and the system (2) has bounded H_∞_ performance.*

**Lemma** **2.** *The RBFNN can approximate any nonlinear function $\bar{f}\left(Z\right)$ defined on a compact set:*

(7) $$f\left(Z\right)=W^{*T}S\left(Z\right)+\delta \left(Z\right)$$

*where $W^{*T}$ is the ideal weight vector, S(Z) is the basic vector, and $\delta \left(Z\right)$ is the approximation error. In addition, there exists an unknown constant ϵ such that $\left|\delta \right|\leq \varepsilon$. ε is defined to prove the bounded stability of the system, and it has no physical meaning in the real world.*

**Lemma** **3** (Young’s inequality [32])**.** *For any $x,y\in R^{2}$, the following inequality holds:*

(8) $$xy\leq \frac{\delta^{p}}{p}{\left|x\right|}^{p}+\frac{1}{q\delta^{q}}{\left|y\right|}^{q}$$

*where δ>0, p>1, q>1, and (p−1)(q−1)=1.*

### 2.3. Prescribed Performance Control

The constrained variable $X_{1}$ is subject to the following prescribed performance constraint:

(9) $$-\rho_{2}<X_{1}<\rho_{1}$$

where $\rho_{1},\rho_{2}$ are the prescribed performance function defined as follows (i = 1, 2):

(10) $$\begin{array}{c} \rho_{i}\left(t\right)=\left\{\begin{array}{c} \rho_{i0}\left(1-\frac{t}{T_{a}}\right)e^{\left(1-\frac{T_{a}}{T_{a}-t}\right)}+\rho_{i\infty },t<T_{a}\\ \rho_{i\infty },t\geq T_{a} \end{array}\right. \end{array}$$

where $\rho_{i0}$, $\rho_{i\infty }$, and $T_{a}$ are the design parameters. $\rho_{i\infty }$ represents the desired steady-state error, $T_{a}$ is the settling time, where $T_{a}>T_{b}$, and $\rho_{i0}$ is typically set 10 to 100 times larger than that of $\rho_{i\infty }$.

To simplify the design process, multiply all three sides of the inequality (9) by 2, then add $-\rho_{2}\left(t\right)+\rho_{1}\left(t\right)$ to each term. Thus, the asymmetric constraint in (9) is transformed into a symmetric constraint:

$$\begin{array}{c} -2\rho_{2}<2X_{1}<2\rho_{1}\\ -\rho_{1}-\rho_{2}<2X_{1}+\rho_{2}-\rho_{1}<\rho_{1}+\rho_{2} \end{array}$$

(11) $$-\left(\rho_{1}\left(t\right)+\rho_{2}\left(t\right)\right)<z\left(t\right)<\rho_{1}\left(t\right)+\rho_{2}\left(t\right)$$

where

(12) $$z\left(t\right)=2X_{1}\left(t\right)+\rho_{2}\left(t\right)-\rho_{1}\left(t\right).$$

A nonlinear mapping is applied to transform z(t) into a smaller and known range as follows:

(13) $$\lambda \left(z\left(t\right)\right)=\tanh\left(z\left(t\right)\right)=\frac{e^{z(t)}-e^{-z(t)}}{e^{z(t)}+e^{-z(t)}}$$

**Remark** **1.** *tanh(·)∈(0,1) is monotonic.*

Similar to (13), we have

(14) $$\lambda \left(\rho_{1}\left(t\right)+\rho_{2}\left(t\right)\right)=\tanh\left(\rho_{1}\left(t\right)+\rho_{2}\left(t\right)\right).$$

Therefore, the constraint design in (11) can be mapped as follows:

(15) $$\lambda \left(-\left(\rho_{1}\left(t\right)+\rho_{2}\left(t\right)\right)\right)<\lambda \left(z\left(t\right)\right)<\lambda \left(\rho_{1}\left(t\right)+\rho_{2}\left(t\right)\right)$$

Consider an indirect constraint function:

(16) $$\varpi \left(t\right)=v\left(t\right)+\lambda (\rho_{1}\left(t\right)+\rho_{2}\left(t\right))$$

where

(17) $$\begin{array}{c} v\left(t\right)=\left\{\begin{array}{c} \left(1-\frac{t}{T_{f}}\right)e^{\left(1-\frac{T_{f}}{T_{f}-t}\right)},t\leq T_{f}\\ 0,t>T_{f} \end{array}\right. \end{array}$$

where $T_{f}$ is a design parameter, representing the time at which the constrained variable enters the prescribed performance region. In addition, let v(t) be referred to as the initial expansion function (IEF).

**Remark** **2.** *The input should be recovered before the IEF changes to 0. In addition, the initial expansion should be finished before the settling time. Thus, the time parameters should be chosen as $T_{b}<T_{f}<T_{a}$.*

Therefore, Theorem 1 can be obtained:

**Theorem** **1.** *When $t>T_{f}$, the control of $\rho_{i}\left(t\right)$ over $X_{1}\left(t\right)$ is equivalent to the control of ϖ(t) over λ(z(t)).*

**Proof** **of Theorem 1.** Based on the monotonicity of the mapping function, it follows that if (15) holds, then (9) also holds. □

To design the BLF and the self-adaptive laws, the coordinate transformations (18) are introduced:

(18) $$\begin{array}{cc} \; & \xi_{1}=\tan\left(\frac{\pi \lambda }{2\varpi }\right) \end{array}$$

(19) $$\begin{array}{cc} \; & \xi_{i}=X_{i}-\alpha_{i-1} \end{array}$$

**Remark** **3.** *In conventional tangent-type BLF methods, the BLF is designed as*

(20) $$\xi_{1}=\tan\left(\frac{\pi z}{2\rho_{i}}\right).$$

*If $\rho_{i0}$ is too large, the constrained variable z may fluctuate within an over large range, which will result in a big overshoot.*

**Remark** **4.** *To constrain λ(z(t)) using the PPC method, the condition λ(z(0))<ϖ(0) must be satisfied. When t=0, $\varpi \left(0\right)=1+\lambda (\rho_{1}\left(0\right)+\rho_{2}\left(0\right))$, even if $X_{1}\left(0\right)$ is not within the range of $\rho_{i}$, λ(z(t)) can still be constrained by ϖ(t).*

Furthermore, one has

(21) $$\begin{array}{cc} {\dot{\xi }}_{1} & =\frac{\pi }{2\cos^{2}\left(\frac{\pi \lambda }{2\varpi }\right)}\left(\frac{\dot{\lambda }\varpi -\lambda \dot{\varpi }}{\varpi^{2}}\right)\\ \; & =\frac{\pi }{2\cos^{2}\left(\frac{\pi \lambda }{2\varpi }\right)}\left(\frac{1}{\varpi }\right)\left(\left(\frac{\partial \lambda }{\partial z}\right)\dot{z}-\frac{\lambda \dot{\varpi }}{\varpi }\right)\\ \; & =R\left(\frac{o_{1}}{o_{2}}\left(\xi_{2}+\alpha_{1}\right)+\frac{{\dot{o}}_{1}}{o_{1}}X_{1}+\frac{1}{2}\left({\dot{\rho }}_{2}-{\dot{\rho }}_{1}\right)-{\left(\frac{2\partial \lambda }{\partial z}\right)}^{-1}\left(\frac{\lambda \dot{\varpi }}{\varpi }\right)\right)\\ {\dot{\xi }}_{i} & ={\dot{X}}_{i}-{\dot{\alpha }}_{i-1}\\ \; & ={\dot{o}}_{i}\frac{X_{i}}{o_{i}}+o_{i}{\dot{x}}_{i}-{\dot{\alpha }}_{i-1} \end{array}$$

where $R=\frac{\pi }{\varpi \cos^{2}\left(\frac{\pi \lambda }{2\varpi }\right)}\frac{\partial \lambda }{\partial z}.$

## 3. Controller Design and Stability Analysis

**Figure 3:**
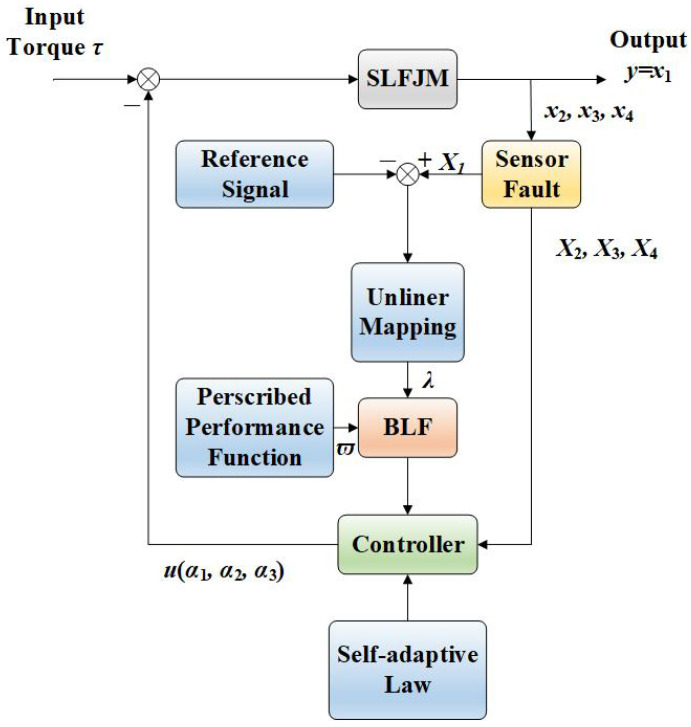
Control block diagram of the system.

The control block diagram of the system is shown in Figure 3. The virtual control laws $\alpha_{i}\left(i=1,2,3\right)$ and actual control law *u* are defined as

(22) $$\begin{array}{cc} \; & \alpha_{1}=-\xi_{1}\eta \left(\frac{c_{1}}{R}+\frac{1}{2b_{1}}{\hat{\beta }}_{1}R\right) \end{array}$$

(23) $$\begin{array}{cc} \; & \alpha_{i}=-\xi_{i}\eta \left(c_{i}+\frac{{\hat{\beta }}_{i}}{2b_{i}}+\frac{1}{4\gamma }\right)\left(i=2,3\right) \end{array}$$

(24) $$\begin{array}{cc} \; & u=-\xi_{4}\eta \left(c_{4}+\frac{{\hat{\beta }}_{4}}{2b_{4}}+\frac{1}{4\gamma }\right) \end{array}$$

where $c_{i},b_{i}\left(i=1,2,3,4\right)$ are designed as positive parameters. γ denotes a disturbance attenuation coefficient, and 0<γ<1. ${\hat{\beta }}_{i}$ is the estimation of $\beta_{i}$. $\beta_{i}$ is defined as

(25) $$\beta_{i}=\frac{N_{i}}{b_{m}}{∥W_{i}^{*}∥}^{2},i=1,2,3,4$$

where ${∥W_{i}^{*}∥}^{2}$ is the ideal weight vector of the *i*th RBFNN, and *N* is the number of its node. In addition, ${\tilde{\beta }}_{i}$ is the estimation error.

Therefore, ${\tilde{\beta }}_{i}$ is calculated by ${\tilde{\beta }}_{i}=\beta_{i}-{\hat{\beta }}_{i}$. In addition, the basis vector is $Z_{i}=\left[X_{1},X_{2},\ldots X_{i-1},y_{d},{\dot{y}}_{d},{\ddot{y}}_{d},\ldots ,{y_{d}}^{\left(i-1\right)},\rho_{1},{\dot{\rho }}_{1},\ldots ,{\rho_{1}}^{\left(i-1\right)},\rho_{2},{\dot{\rho }}_{2},\ldots ,{\rho_{2}}^{\left(i-1\right)},\eta ,\dot{\eta },\ldots ,\eta^{\left(i-2\right)}\right].$

The adaptive laws are defined as

(26) $$\begin{array}{cc} \; & {\dot{\hat{\beta }}}_{1}=\frac{1}{2b_{1}}\xi_{1}^{2}R^{2}\eta -\mu_{1}{\hat{\beta }}_{1} \end{array}$$

(27) $$\begin{array}{cc} \; & {\dot{\hat{\beta }}}_{i}=\frac{1}{2b_{i}}\xi_{i}^{2}\eta -\mu_{i}{\hat{\beta }}_{i},\left(i=2,3,4\right) \end{array}$$

where $\mu_{i}$ are positive parameters.

**Remark** **5.** *The $\xi_{1}^{2}$ means ${\left(\xi_{1}\right)}^{2}$. In the following text, the ${\tilde{\beta }}_{1}^{2}$ means ${\left({\tilde{\beta }}_{1}\right)}^{2}$.*

Step (1) is defined as follows:

Define the Lyapunov function $V_{1}$ as

(28) $$V_{1}=\frac{1}{2}\xi_{1}^{2}+\frac{1}{2}b{\tilde{\beta }}_{1}^{2}.$$

Then, the derivative of $V_{1}$ is

(29) $$\begin{array}{cc} {\dot{V}}_{1} & =\xi_{1}{\dot{\xi }}_{1}-b{\tilde{\beta }}_{1}{\dot{\hat{\beta }}}_{1}\\ \; & =\xi_{1}R\left(\frac{o_{1}}{o_{2}}\left(\xi_{2}+\alpha_{1}\right)+\frac{{\dot{o}}_{1}}{o_{1}}X_{1}+\frac{1}{2}\left({\dot{\rho }}_{2}-{\dot{\rho }}_{1}\right)-{\left(\frac{2\partial \lambda }{\partial z}\right)}^{-1}\left(\frac{\lambda \dot{\varpi }}{\varpi }\right)\right)-b{\tilde{\beta }}_{1}{\dot{\hat{\beta }}}_{1}. \end{array}$$

Using Young’s inequality, we have

(30) $$\xi_{1}R\frac{o_{1}}{o_{2}}\xi_{2}\leq \frac{1}{2}\xi_{1}^{2}R^{2}\frac{{o_{1}}^{2}}{{o_{2}}^{2}}+\frac{1}{2}\xi_{2}^{2}.$$

Combining (29) and (30), we have

(31) $${\dot{V}}_{1}\leq \xi_{1}R\left({\bar{f}}_{1}\left(Z_{1}\right)+\alpha_{1}\right)+\frac{1}{2}{\xi_{2}}^{2}-b{\tilde{\beta }}_{1}{\dot{\hat{\beta }}}_{1}-\frac{1}{2b_{1}}b\xi_{1}^{4}R^{4}-\frac{1}{2}\xi_{1}^{2}R^{2}$$

where

(32) $$\begin{array}{c} {\bar{f}}_{1}\left(Z_{1}\right)=\frac{1}{2}\frac{{o_{1}}^{2}}{{o_{2}}^{2}}\xi_{1}R+\frac{{\dot{o}}_{1}}{o_{1}}X_{1}+\frac{1}{2}\left({\dot{\rho }}_{2}-{\dot{\rho }}_{1}\right)-{\left(\frac{2\partial \lambda }{\partial z}\right)}^{-1}\left(\frac{\lambda \dot{\varpi }}{\varpi }\right)+\frac{1}{2}\xi_{1}R+\frac{1}{2b_{1}}b\xi_{1}^{3}R^{3}. \end{array}$$

${\bar{f}}_{1}\left(Z_{1}\right)$ can be approximated using the following RBFNN:

(33) $${\bar{f}}_{1}\left(Z_{1}\right)={W_{1}}^{*T}S\left(Z_{1}\right)+\delta \left(Z_{1}\right)$$

where $Z_{1}=\left[x_{1},{{\bar{y}}_{d}}^{\left(1\right)},{\bar{\rho }}_{1}^{\left(1\right)},{\bar{\rho }}_{2}^{\left(1\right)},{\bar{\varpi }}^{\left(1\right)}\right]$.

Based on Young’s inequality and (25), we have

(34) $$\begin{array}{cc} \xi_{1}R{\bar{f}}_{1}\left(Z_{1}\right) & =\xi_{1}RW_{1}^{*T}S_{1}\left(Z_{2}\right)+\xi_{1}R\delta_{1}\left(Z_{1}\right)\\ \; & =\frac{1}{2b_{1}}\xi_{1}^{2}R^{2}W_{1}^{*T}W_{1}S_{1}^{T}S_{1}+\frac{1}{2}b_{1}+\frac{1}{2}\xi_{1}^{2}R^{2}+\frac{1}{2}\varepsilon_{1}^{2}\\ \; & =\frac{1}{2b_{1}}b\xi_{1}^{2}R^{2}\beta_{1}+\frac{1}{2}b_{1}+\frac{1}{2}\xi_{1}^{2}R^{2}+\frac{1}{2}\varepsilon_{1}^{2}\\ \; & =\frac{1}{2b_{1}}b\xi_{1}^{2}R^{2}\beta_{1}+\frac{1}{2b_{1}}b\xi_{1}^{2}R^{2}\eta \beta_{1}+\frac{1}{2}b_{1}+\frac{1}{2}\xi_{1}^{2}R^{2}+\frac{1}{2}\varepsilon_{1}^{2}\\ \; & =\frac{1}{2b_{1}}b\xi_{1}^{4}R^{4}+\frac{1}{2b_{1}}b\beta_{1}^{2}+\frac{1}{2b_{1}}b\xi_{1}^{2}R^{2}\eta \beta_{1}+\frac{1}{2}b_{1}+\frac{1}{2}\xi_{1}^{2}R^{2}+\frac{1}{2}\varepsilon_{1}^{2}. \end{array}$$

Then, from (26), we obtain

(35) $$-b{\tilde{\beta }}_{1}{\dot{\hat{\beta }}}_{1}=-b{\tilde{\beta }}_{1}\left(\frac{1}{2b_{1}}\xi_{1}^{2}R^{2}\eta -\mu_{1}{\hat{\beta }}_{1}\right)$$

where

(36) $$\begin{array}{cc} b\mu_{1}{\tilde{\beta }}_{1}{\hat{\beta }}_{1} & =b\mu_{1}{\tilde{\beta }}_{1}\beta_{1}-{\tilde{\beta }}_{1}\\ \; & =-b\mu_{1}{\tilde{\beta }}_{1}^{2}+b\mu_{1}{\tilde{\beta }}_{1}\beta_{1}\\ \; & \leq -b\mu_{1}{\tilde{\beta }}_{1}^{2}+\frac{1}{2}b\mu_{1}{{\tilde{\beta }}_{1}}^{2}+\frac{1}{2}b\mu_{1}\beta_{1}^{2}\\ \; & \;\;\;\;\;\;=-\frac{1}{2}b\mu_{1}{\tilde{\beta }}_{1}^{2}+\frac{1}{2}b\mu_{1}\beta_{1}^{2} \end{array}$$

Invoking (32), (34), (35) and (36), we have

(37) $$\begin{array}{cc} {\dot{V}}_{1} & \leq -c_{1}b\eta \xi_{1}^{2}-\frac{1}{2}b\mu_{1}{{\tilde{\beta }}_{1}}^{2}+\frac{1}{2}\xi_{2}^{2}+\frac{1}{2}b\mu_{1}\beta_{1}^{2}+\frac{1}{2b_{1}}b\beta_{1}^{2}+\frac{1}{2}b_{1}+\frac{1}{2}\varepsilon_{1}^{2}\\ \; & \leq -p_{1}\eta V_{1}+\frac{1}{2}\xi_{2}^{2}+q_{1} \end{array}$$

where $p_{1}=\min\left\{2bc_{1},2\mu_{1}\right\}$, $q_{1}=\frac{1}{2}b_{m}\mu_{1}\beta_{1}^{2}+\frac{1}{2b_{1}}b_{m}\beta_{1}^{2}+\frac{1}{2}b_{1}+\frac{1}{2}\varepsilon_{1}^{2}$. $p_{i}$ and $q_{i}$ are defined to prove the bounded stability of the system. They have no physical meaning in the real world.

Step (2) is defined as follows:

Define the Lyapunov function $V_{2}$ as

(38) $$V_{2}=\frac{1}{2}\xi_{2}^{2}+\frac{1}{2}b{\tilde{\beta }}_{2}^{2}$$

The derivative of $V_{2}$ is given by

(39) $$\begin{array}{cc} {\dot{V}}_{2} & =\xi_{2}{\dot{\xi }}_{2}-b{\tilde{\beta }}_{2}{\dot{\hat{\beta }}}_{2}\\ \; & =\xi_{2}\left(\frac{{\dot{o}}_{2}}{o_{2}}X_{2}+\frac{o_{2}}{o_{3}}g_{2}\xi_{3}+\alpha_{2}\right)+o_{2}f_{2}+o_{2}d_{2}-{\dot{\alpha }}_{1})-b{\tilde{\beta }}_{2}{\dot{\hat{\beta }}}_{2} \end{array}$$

Using Young’s inequality, we derive

(40) $$\begin{array}{cc} \; & \frac{o_{2}}{o_{3}}\xi_{2}g_{2}\xi_{3}\leq \frac{1}{2}\frac{o_{2}^{2}}{{o_{3}}^{2}}g_{2}^{2}\xi_{2}^{2}+\frac{1}{2}\xi_{3}^{2} \end{array}$$

(41) $$\begin{array}{cc} \; & o_{2}\xi_{2}d_{2}\leq \frac{1}{4\gamma }o_{2}^{2}{\xi_{2}}^{2}+\frac{1}{4\gamma }\eta o_{2}^{2}\xi_{2}^{2}+\gamma {d_{2}}^{2} \end{array}$$

Invoking (39), (41) and (40), we have

(42) $$\begin{array}{cc} {\dot{V}}_{2}\leq & \xi_{2}\left({\bar{f}}_{2}\left(Z_{2}\right)+\frac{o_{2}}{o_{3}}g_{2}\alpha_{2}\right)+\frac{1}{2}\xi_{3}^{2}+\gamma d_{2}^{2}-b{\tilde{\beta }}_{2}{\dot{\hat{\beta }}}_{2}-\xi_{2}^{2}-\frac{b_{m}}{2b_{2}}\xi_{2}^{4} \end{array}$$

where

(43) $$\begin{array}{cc} {\bar{f}}_{2}\left(Z_{2}\right)= & \frac{{\dot{o}}_{2}}{o_{2}}X_{2}+\frac{1}{2}\frac{o_{2}^{2}}{o_{3}^{2}}{g_{2}}^{2}\xi_{2}+o_{2}f_{2}+\left(1+\frac{1}{4\gamma }o_{2}^{2}\right)\xi_{2}-{\dot{\alpha }}_{1}+\frac{b_{m}}{2b_{2}}\xi_{2}^{3} \end{array}$$

${\bar{f}}_{2}\left(Z_{2}\right)$ can be approximated by the following RBFNN:

(44) $${\bar{f}}_{2}\left(Z_{2}\right)=W_{2}^{*T}S_{2}\left(Z_{2}\right)+\delta_{2}\left(Z_{2}\right)$$

in which $\left|\delta_{2}\left(Z_{2}\right)\right|<\varepsilon_{2}$.

As with (36), we obtain

(45) $$\begin{array}{cc} \; & \frac{o_{2}}{o_{3}}\xi_{2}g_{2}\alpha_{2}-b{\tilde{\beta }}_{2}{\dot{\hat{\beta }}}_{2}+\frac{1}{4\gamma }\eta o_{2}^{2}\xi_{2}^{2}\leq b\left(-c_{2}\eta \xi_{2}^{2}-\frac{1}{2b_{2}}\eta \xi_{2}^{2}{\hat{\beta }}_{2}\right)-b{\tilde{\beta }}_{2}\left(\frac{1}{2b_{2}}\eta \xi_{2}^{2}-\mu_{2}{\hat{\beta }}_{2}\right). \end{array}$$

Using Young’s inequality, we can obtain

(46) $$\begin{array}{cc} \; & b\mu_{2}{\tilde{\beta }}_{2}{\hat{\beta }}_{2}\leq -\frac{1}{2}b\mu_{2}{\tilde{\beta }}_{2}^{2}+\frac{1}{2}b\mu_{2}\beta_{2}^{2}\\ \; & \xi_{2}{\bar{f}}_{2}\left(Z_{2}\right)\leq \frac{1}{2b_{2}}b\xi_{2}^{4}+\frac{1}{2b_{2}}b\beta_{2}^{2}+\frac{1}{2b_{2}}b\eta \beta_{2}\xi_{2}^{2}+\frac{1}{2}b_{2}+\frac{1}{2}\xi_{2}^{2}+\frac{1}{2}\varepsilon_{2}^{2} \end{array}$$

Invoking (42), (45) and (46), we have

(47) $$\begin{array}{cc} {\dot{V}}_{2} & \leq -c_{2}b\eta \xi_{2}^{2}-\frac{1}{2}b\mu_{2}{\tilde{\beta }}_{2}^{2}+\frac{1}{2}\xi_{3}^{2}-\frac{1}{2}\xi_{2}^{2}+\frac{1}{2b_{2}}b\beta_{2}^{2}+\frac{1}{2}b\mu_{2}\beta_{2}^{2}+\frac{1}{2}b_{2}+\frac{1}{2}\varepsilon_{2}^{2}+\frac{1}{2\gamma }d_{2}^{2}\\ \; & \leq -p_{2}\eta V_{2}+\frac{1}{2}\xi_{3}^{2}-\frac{1}{2}\xi_{2}^{2}+q_{2} \end{array}$$

where $p_{2}=\min\left\{2bc_{2},2\mu_{2}\right\}$, $q_{2}=\frac{1}{2b_{2}}b_{m}\beta_{2}^{2}+\frac{1}{2}b_{m}\mu_{2}\beta_{2}^{2}+\frac{1}{2}b_{2}+\frac{1}{2}\varepsilon_{2}^{2}+\gamma d_{2}^{2}$.

Step (3) is defined as follows:

Define the Lyapunov function $V_{3}$ as

(48) $$V_{3}=\frac{1}{2}\xi_{3}^{2}+\frac{1}{2}b{{\tilde{\beta }}_{3}}^{2}$$

The derivative of $V_{3}$ is

(49) $$\begin{array}{cc} {\dot{V}}_{3} & =\xi_{3}{\dot{\xi }}_{3}-b{\tilde{\beta }}_{3}{\dot{\hat{\beta }}}_{3}\\ \; & =\xi_{3}(\frac{{\dot{o}}_{3}}{o_{3}}X_{3}+\frac{o_{3}}{o_{4}}\left(\xi_{4}+\alpha_{3}-{\dot{\alpha }}_{2}\right)-b{\tilde{\beta }}_{3}{\dot{\hat{\beta }}}_{3}. \end{array}$$

Using Young’s inequality, we obtain

(50) $$\frac{o_{3}}{o_{4}}\xi_{3}\xi_{4}\leq \frac{1}{2}\frac{{o_{3}}^{2}}{{o_{4}}^{2}}\xi_{3}^{2}+\frac{1}{2}\xi_{4}^{2}.$$

Combining (49) and (50), we obtain

(51) $${\dot{V}}_{3}\leq \xi_{3}\left(\alpha_{3}+{\bar{f}}_{3}\left(Z_{3}\right)\right)+\frac{1}{2}\xi_{4}^{2}-b{\tilde{\beta }}_{3}{\dot{\hat{\beta }}}_{3}-\xi_{3}^{2}$$

where ${\bar{f}}_{3}\left(Z_{3}\right)=\frac{{\dot{o}}_{3}}{o_{3}}X_{3}+\left(\frac{{o_{3}}^{2}}{{o_{4}}^{2}}+1\right)\xi_{3}-{\dot{\alpha }}_{2}+\frac{1}{2b_{3}}\xi_{3}^{4}$.

${\bar{f}}_{3}\left(Z_{3}\right)$ can be approximated by the following RBFNN:

(52) $${\bar{f}}_{3}\left(Z_{3}\right)=W_{3}^{{*}^{T}}S_{3}\left(Z_{3}\right)+\delta_{3}\left(Z_{3}\right)$$

where $\left|\delta_{3}\left(Z_{3}\right)\right|\leq \varepsilon_{3}$.

Similar to (40) and (41), one has

(53) $$\begin{array}{cc} \; & \xi_{3}{\bar{f}}_{3}\left(Z_{3}\right)\leq \frac{1}{2b_{3}}b\xi_{3}^{4}+\frac{1}{2b_{3}}b\eta \beta_{3}\xi_{3}^{2}+\frac{1}{2b_{3}}b\beta_{3}^{2}+\frac{1}{2}b_{3}+\frac{1}{2}\xi_{3}^{2}+\frac{1}{2}\varepsilon_{3}^{2} \end{array}$$

(54) $$\begin{array}{cc} \; & \xi_{3}\alpha_{3}-b{\tilde{\beta }}_{3}{\dot{\hat{\beta }}}_{3}\leq -c_{3}b\eta \xi_{3}^{2}-\frac{1}{2b_{3}}b\eta \xi_{3}^{2}{\hat{\beta }}_{3}-\frac{1}{2b_{3}}b\eta \xi_{3}^{2}{\tilde{\beta }}_{3}+-\frac{1}{2}b\mu_{3}{\tilde{\beta }}_{3}^{2}+\frac{1}{2}b\mu_{3}\beta_{3}^{2} \end{array}$$

Invoking (51), (53) and (54), we have

(55) $$\begin{array}{cc} {\dot{V}}_{3} & \leq -c_{3}b\eta \xi_{3}^{2}-\frac{1}{2}\mu_{3}b{\tilde{\beta }}_{3}^{2}+\frac{1}{2}\xi_{4}^{2}-\frac{1}{2}\xi_{3}^{2}+\frac{1}{2}b_{3}+\frac{1}{2}\varepsilon_{3}^{2}+\frac{1}{2b_{3}}b\beta_{3}^{2}+\frac{1}{2}b\mu_{3}\beta_{3}^{2}\\ \; & \leq -p_{3}\eta V_{3}+\frac{1}{2}\xi_{4}^{2}-\frac{1}{2}\xi_{3}^{2}+q_{3} \end{array}$$

where $p_{3}=\min\left\{2bc_{3},2\mu_{3}\right\}$, $q_{3}=\frac{1}{2b_{3}}b\beta_{3}^{2}+\frac{1}{2}b\mu_{3}\beta_{3}^{2}+\frac{1}{2}b_{3}+\frac{1}{2}\varepsilon_{3}^{2}$.

Step (4) is defined as follows:

Choose a Lyapunov function $V_{4}$ as

(56) $$V_{4}=\frac{1}{2}\xi_{4}^{2}+\frac{1}{2}b{\tilde{\beta }}_{4}^{2}$$

Different from (56), we have

(57) $${\dot{V}}_{4}=\xi_{4}\left(\frac{{\dot{o}}_{4}}{o_{4}}X_{4}+o_{4}g_{4}u+o_{4}f_{4}+o_{4}d_{4}-{\dot{\alpha }}_{3}\right)-b{\tilde{\beta }}_{4}{\dot{\hat{\beta }}}_{4}$$

Applying Young’s inequality yields

(58) $$\xi_{4}d_{4}\leq \frac{1}{4\gamma }\xi_{4}^{2}+\gamma d_{4}^{2}+\frac{1}{4\gamma }\eta \xi_{4}^{2}$$

one has

(59) $${\dot{V}}_{4}\leq \xi_{4}\left(g_{4}u+{\bar{f}}_{4}\left(Z_{4}\right)+\frac{1}{4\gamma }\eta \xi_{4}\right)-b{\tilde{\beta }}_{4}{\dot{\hat{\beta }}}_{4}+\gamma d_{4}^{2}-\xi_{4}^{2}-\frac{1}{2b_{4}}b_{m}\xi_{4}^{3}$$

where ${\bar{f}}_{4}\left(Z_{4}\right)=\frac{{\dot{o}}_{4}}{o_{4}}X_{4}+o_{4}f_{4}+\left(\frac{1}{4\gamma }+1\right)\xi_{4}+\frac{1}{2b_{4}}b\xi_{4}^{3}-{\dot{\alpha }}_{3}$.

Similarly, ${\bar{f}}_{4}\left(Z_{4}\right)$ can be approximated as follows:

(60) $${\bar{f}}_{4}\left(Z_{4}\right)=W_{4}^{*T}S_{4}\left(Z_{4}\right)+\delta_{4}\left(Z_{4}\right)$$

where $\left|\delta_{4}\left(Z_{4}\right)\right|\leq \varepsilon_{4}$.

Following the similar lines in (53) and (54), we obtain

(61) $$\begin{array}{cc} \; & \xi_{4}{\bar{f}}_{4}\left(Z_{4}\right)\leq \frac{1}{2b_{4}}b_{m}\xi_{4}^{4}+\frac{1}{2b_{4}}b_{m}\eta \beta_{4}+\frac{1}{2b_{4}}b_{m}\beta_{4}^{2}+\frac{1}{2}b_{4}+\frac{1}{2}\xi_{4}^{2}+\frac{1}{2}\varepsilon_{4}^{2} \end{array}$$

(62) $$\begin{array}{cc} \; & \xi_{4}g_{4}u-b{\hat{\beta }}_{4}{\dot{\hat{\beta }}}_{4}+\frac{1}{4\gamma }\eta \xi_{4}^{2}\\ \; & \leq -c_{4}b\eta \xi_{4}^{2}-\frac{1}{2b_{4}}b\eta \xi_{4}^{2}{\hat{\beta }}_{4}-\frac{1}{2b_{4}}b\eta \xi_{4}^{2}{\tilde{\beta }}_{4}+-\frac{1}{2}b\mu_{4}{\tilde{\beta }}_{4}^{2}+\frac{1}{2}b\mu_{4}\beta_{4}^{2} \end{array}$$

Inserting (61) and (62) into (59) yields

(63) $$\begin{array}{cc} {\dot{V}}_{4} & \leq -bc_{4}\eta \xi_{4}^{2}-\frac{1}{2}b\mu_{4}{\tilde{\beta }}_{4}^{2}+\frac{1}{2}b\mu_{4}\beta_{4}^{2}+\frac{1}{2}b\beta_{4}^{2}+\frac{1}{2}b_{4}-\frac{1}{2}\xi_{4}^{2}+\frac{1}{2}\varepsilon_{4}^{2}+\gamma d_{4}^{2}\\ \; & \leq -p_{4}\eta V_{4}+q_{4}-\frac{1}{2}\xi_{4}^{2} \end{array}$$

where $p_{4}=\min\left\{2bc_{4},2\mu_{4}\right\}$,$q_{4}=\frac{1}{2}b\mu_{4}\beta_{4}^{2}+\frac{1}{2}b\beta_{4}^{2}+\frac{1}{2}b_{4}+\frac{1}{2}\varepsilon_{4}^{2}+\gamma d_{4}^{2}$.

**Theorem** **2.** *Consider the SLFJM system (1) with a sensor fault under Assumption 1. If the virtual control laws and actual control laws are given in (22)–(24) and the adaptive laws are given in (26) and (27), then we have the following:*

*1.*  *The output remains within the prescribed performance boundaries specified by the PFTPF when $T>T_{a}$.*
*2.*  *The system exhibits H_∞_ disturbance attenuation performance for external disturbances.*
*3.*  *All signals in the system are bounded.*

**Proof** **of Theorem 2.** Define the Lyapunov function *V* of the SLFJM system as

(64) $$V=\sum_{i=1}^{4}V_{i}$$

From (37), (47), (55) and (63), it can be concluded that

(65) $$\dot{V}\leq -p\eta V+q$$

where $p=\min\left\{2b_{m}c_{i},2\mu_{i}\right\}$, $q=\sum_{i=1}^{4}q_{i}$.

1. Proof of prescribed performance:
  According to (65) and Lemma (1), it follows that $\xi_{i}$ is bounded. Furthermore, because $\xi_{1}\in I_{\infty }$ and λ(0)<ϖ(0) as well as λ(t)<ϖ(t), the prescribed performance is achieved.
  Furthermore, the output of the SLFJM enters the range $\left(-\rho_{2},\rho_{1}\right)$ when $t>T_{f}$ and the range $\left(-\rho_{1\infty }\left(t\right),\rho_{2\infty }\left(t\right)\right)$ when $t\geq T_{a}$. Thus, the prescribed performance is guaranteed.
2. Stability analysis:
  Because of the continuity of the output signal, $X_{1}$ is bounded. Consequently, $x_{1}$ and *z* are bounded. Similarly, $x_{i}$ is bounded.
  Furthermore, from the control laws, ${\hat{\beta }}_{1}\in I_{\infty }$. Thus, ${\hat{\beta }}_{1}$ is bounded.
  According to (19), it follows that $\alpha_{i}$ is bounded. Similarly, *u* is bounded.
  This completes the stability analysis of the system.
3. Proof of bounded-H_∞_ disturbance attenuation performance:
  Define an auxiliary Lyapunov function as
  (66) $$\bar{V}=V+\phi$$
  where ϕ is a positive constant applied in H_∞_ disturbance attenuation performance. ϕ is introduced to ensure that $\bar{V}>0$. The boundness of the system shows that the number ϕ exists.
  Define an auxiliary function
  (67) $$H=\dot{\bar{V}}+2\xi_{1}^{2}\left(X_{1}^{2}+1\right)-\gamma {∥d∥}^{2}$$
  where ${∥d∥}^{2}=\sum_{i=1}^{4}d_{i}^{2}$.
  Invoking (67) into (65), we can obtain a positive constant *h*, ensuring
  (68) $$H\leq q_{0}\leq h\bar{V}$$
  where $q_{0}=\sum_{i=1}^{4}q_{i}-\gamma {∥d_{i}∥}^{2}$. The *h* is defined to prove the bounded-H_∞_ performance of the system. In addition, *h* has no physical meaning in the real world and does not join the designing of the controller.
  In addition,
  (69) $$\dot{\bar{V}}\leq \frac{1}{2\bar{\gamma }}{∥d∥}^{2}+h\bar{V}-2\xi_{1}^{2}\left(s\right)\left(X_{1}^{2}\left(s\right)+1\right)$$
  Integrating (69) from 0 to *t*, one has
  (70) $$\begin{array}{cc} \; & \bar{V}\left(\xi \left(t\right)\right)-\bar{V}\left(\xi \left(0\right)\right)\\ \; & \leq \int_{0}^{t}h\bar{V}\left(\xi \left(s\right)\right)ds+\int_{0}^{t}\left(\frac{1}{2}\gamma {∥d∥}^{2}-2\xi_{1}^{2}\left(s\right)\left(z_{1}^{2}\left(s\right)+1\right)\right)ds. \end{array}$$
  Therefore, (70) can also be expressed as
  (71) $$0<\bar{V}\left(\xi \left(t\right)\right)\leq \Xi \left(t\right)+\int_{0}^{t}h\bar{V}\left(\xi \left(s\right)\right)ds$$
  where $\Xi \left(t\right)=\int_{0}^{t}\left(\frac{1}{2\bar{\gamma }}{∥d∥}^{2}-2\xi_{1}^{2}\left(s\right)\left(z_{1}^{2}\left(s\right)+1\right)\right)ds+\bar{V}\left(\xi \left(0\right)\right)$.
  According to the Gronwall’s inequality, it can be deduced that
  (72) $$\bar{V}\leq \Xi \left(t\right)+\int_{0}^{t}h\Xi \left(s\right)e^{\int_{s}^{t}hdu}ds$$
  Next, we present a proof of contradiction to demonstrate that Ξ(t)>0.
  If Ξ(t)≤0, then
  (73) $$\int_{0}^{t}h\Xi \left(s\right)e^{\int_{s}^{t}hdu}ds\leq 0$$
  This results in a contradiction between (72) and (73).
  Thus, Ξ(t)>0, and we conclude that
  (74) $$\int_{0}^{t}2\xi_{1}^{2}\left(s\right)\left(z_{1}^{2}\left(s\right)+1\right)ds<\int_{0}^{t}\left(\frac{1}{2}\gamma {∥d∥}^{2}\right)ds+\bar{V}\left(\xi \left(0\right)\right)$$
  Applying Gronwall’s inequality and noting that ϖ(0)>ϖ(t), we obtain
  (75) $$\begin{array}{c} \int_{0}^{t}2\xi_{1}^{2}\left(s\right)\left(z_{1}^{2}\left(s\right)+1\right)ds\\ \geq \int_{0}^{t}2{\left(\frac{\pi \tanh(z_{1}\left(t\right))}{2\varpi (0)}\right)}^{2}\left(z_{1}^{2}\left(s\right)+1\right)ds\\ \geq \frac{\pi^{2}}{4}\int_{0}^{t}\frac{z_{1}^{2}}{\varpi {\left(0\right)}^{2}}ds \end{array}$$
  which means that
  (76) $$\int_{0}^{t}z_{1}^{2}ds\leq \int_{0}^{t}\left({\bar{\gamma }}^{2}{∥d∥}^{2}\right)ds+\frac{4\varpi^{2}\left(0\right)}{\pi^{2}}\bar{V}\left(\xi \left(0\right)\right)$$
  where $\bar{\gamma }=\frac{\sqrt{2\gamma }}{\pi }$. Therefore, the system satisfies the performance index defined in Definition 1, completing the proof of Theorem 2.

□

## 4. Simulation

In this section, two stabilization control simulation examples are presented to verify the effectiveness of the controller. In addition, the ODE45 solver was adopted, and the simulation step was set to 0.0001.

In this section, the parameters of the SLFJM have been set as follows. The length of the force arm *L* was set to 1 m. The link mass *M* was set as 2 kg, and the innertia value of the link *I* was defined as 2 kg·m^2^. The inertia value of the motor *J* was assigned as 0.5 kg·m^2^, and the spring constant of the joint stiffness *K* was established as 10 N·m/rad. The gravitational acceleration *g* was taken as 9.8 m/s^2^.

The fault loss functions are modeled as

$$o_{1}=\left\{\begin{array}{c} 1,t<0.2\\ 0.5+0.5e^{-t},t\geq 0.2 \end{array}\right.,o_{2}=\left\{\begin{array}{c} 1,t<0.2\\ e^{-t},t\geq 0.2 \end{array}\right.,$$

$$o_{3}\left(t\right)=\left\{\begin{array}{c} 1,t<0.5\\ 0.75,t\geq 0.5 \end{array}\right.,o_{4}\left(t\right)=\left\{\begin{array}{c} 1,t<0.5\\ 0.5,t\geq 0.5 \end{array}\right..$$

Moreover, the disturbances are given by $d_{1}=0.1\sin\left(t\right)$, $d_{2}=0.1\cos\left(t\right)$.

The controller was designed according to Theorem 2. The parameters were chosen as $b_{1}=b_{2}=b_{3}=b_{4}=50$, $c_{1}=c_{2}=3$, $c_{3}=c_{4}=80$, and $\mu_{1}=\mu_{2}=\mu_{3}=\mu_{4}=0.3$. In addition, we chose the time parameters as $T_{a}=2$, $T_{f}=1.5$, and $T_{b}=1$. Thus, the prescribed performance functions are provided as

$$\rho_{1}\left(t\right)=\left\{\begin{array}{c} 0.6\left(1-\frac{t}{2}\right)e^{\left(1-\frac{2}{2-t}\right)}+0.03\left(t<T_{a}\right)\\ 0.03(t\geq T_{a}), \end{array}\right.,$$

$$\rho_{2}\left(t\right)=\left\{\begin{array}{c} 0.5\left(1-\frac{t}{2}\right)e^{\left(1-\frac{2}{2-t}\right)}+0.03\left(t<T_{a}\right)\\ 0.02(t\geq T_{a}) \end{array}\right..$$

In addition, the IEF v(t) was chosen as

$$v\left(t\right)=\left\{\begin{array}{c} \left(1-\frac{t}{T_{f}}\right)e^{\left(1-\frac{T_{f}}{T_{f}-t}\right)},t\leq 1.5\\ 0,t>1.5 \end{array}\right.,$$

and the ICF η(t) was considered as

$$\eta \left(t\right)=\left\{\begin{array}{c} -\left(1-\frac{t}{T_{b}}\right)e^{\left(1-\frac{T_{b}}{3-T_{b}}\right)}+1,t\leq 1\\ 1,t>1. \end{array}\right.$$

**Figure 4:**
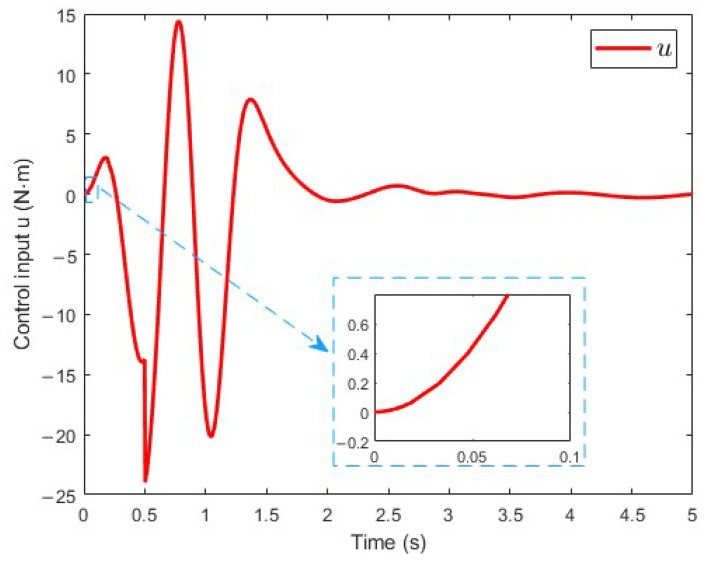
Control input.

**Figure 5:**
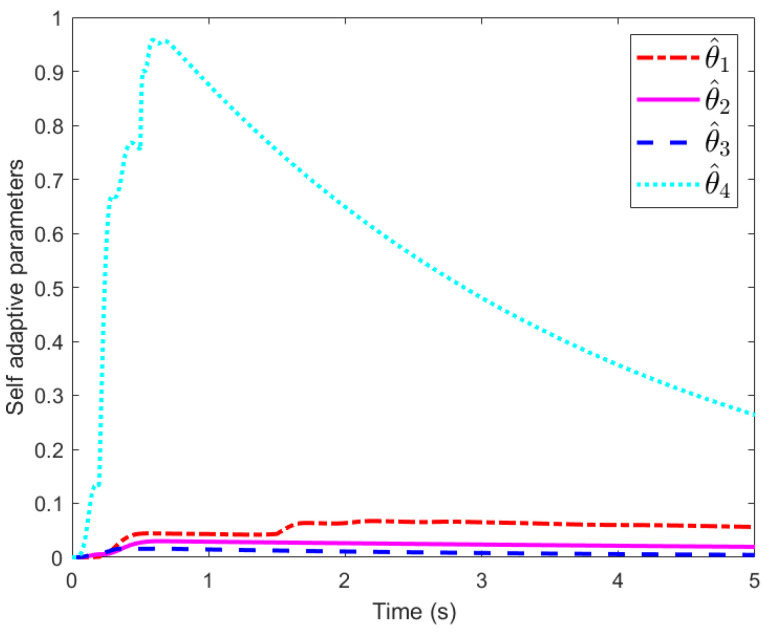
Self-adaptive parameters.

**Figure 6:**
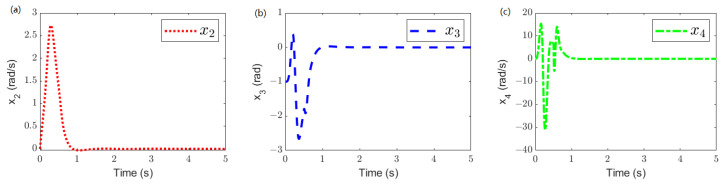
System states: (**a**) $x_{2}$. (**b**) $x_{3}$. (**c**) $x_{4}$.

**Figure 7:**
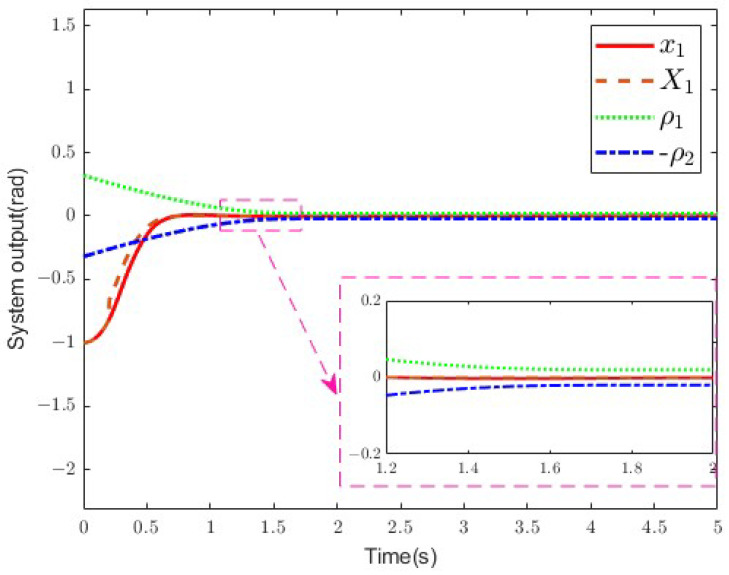
System output.

The simulation results are shown in Figure 4, Figure 5, Figure 6 and Figure 7.

Figure 4 illustrates the system input *u*, which denotes the input torque τ. As observed, the control signal starts from 0. This simulation result indicates that the overlarge step control input is prevented.

The system states $x_{2}$, $x_{3}$, and $x_{4}$ are shown in Figure 6. They represents the link angle speed $\dot{q}$, the motor angle position θ, and the motor angle speed $\dot{\theta }$, respectively. As time progresses, these three states of the SLFJM become bounded.

Figure 5 shows the responses of adaptive parameters ${\hat{\beta }}_{1}$, ${\hat{\beta }}_{2}$, ${\hat{\beta }}_{3}$, and ${\hat{\beta }}_{4}$. These parameters first increase and then converge to a small neighborhood of 0. Thus, we conclude that these states are bounded.

Figure 7 presents the system output *y*, the fault signal $X_{1}$, and the performance bounds $\rho_{1}$ and $-\rho_{2}$. *y* corresponds to the link angle position *q*. As can be seen, the output of the SLFJM becomes stabilized under disturbance. Additionally, the system output remains constrained by the prescribed performance function, even if the initial constrained variable is outside the predefined region.

To demonstrate the adaptability of the proposed method to different initial states, several stabilization simulations were conducted in different initial conditions, as shown in Cases 1–3:

1. Case 1: $[x_{1}\left(0\right),x_{2}\left(0\right),x_{3}\left(0\right),x_{4}\left(0\right)]=\left[1,0,1,0\right]$;
2. Case 2: $[x_{1}\left(0\right),x_{2}\left(0\right),x_{3}\left(0\right),x_{4}\left(0\right)]=\left[2,0,2,0\right]$;
3. Case 3: $[x_{1}\left(0\right),x_{2}\left(0\right),x_{3}\left(0\right),x_{4}\left(0\right)]=\left[-2,0,-2,0\right]$.

**Figure 8:**
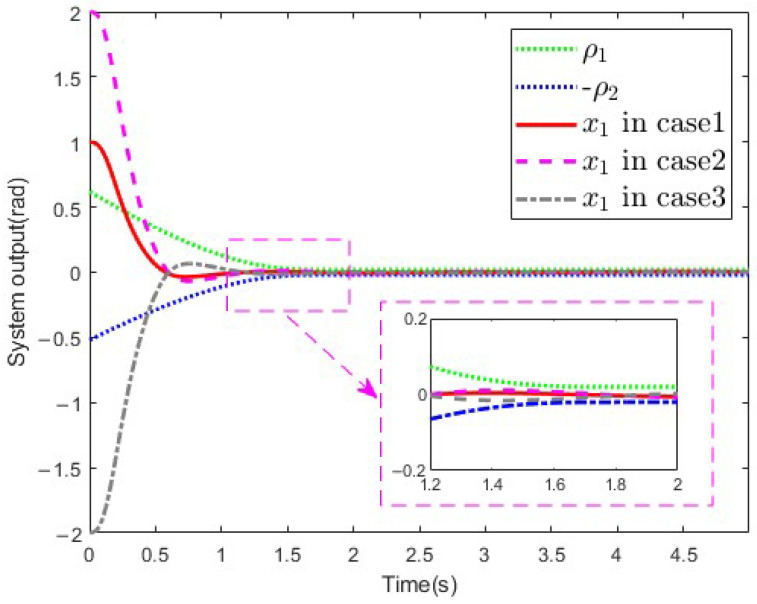
System output in different cases.

In this simulation, except for the parameters of the initial state, all other system parameters remained the same. The system output is shown in Figure 8. As can be seen, the prescribed performance could be achieved under different initial states of the SLMFJ. Therefore, it can be concluded that the control performance is independent of the initial state.

**Figure 9:**
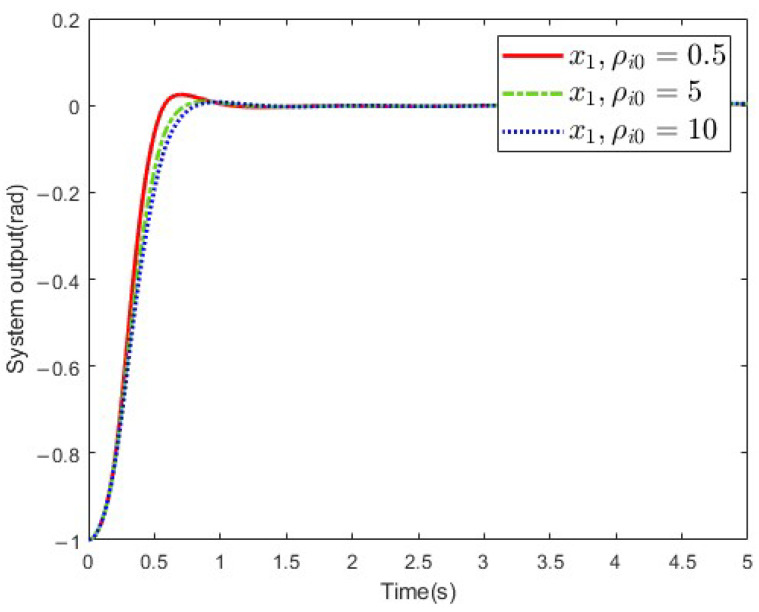
System output with different $\rho_{i0}$ values.

Furthermore, several simulations with various $\rho_{i0}$ values were conducted to study its influence on the control performance. Simulations under the cases $\rho_{i0}=0.2$, $\rho_{i0}=1$, and $\rho_{i0}=2$ were conducted. In the simulation, the other parameters except the prescribed performance function remained the same. The simulation results are shown in Figure 9. It can be concluded from this figure that a better control performance can be achieved by reducing the $\rho_{i0}$.

In order to verify the superiority of the proposed method, a comparative simulation against other conventional methods is provided. The methods are shown as follows:

1. Method proposed in this paper.
2. Logarithm-type BLF method [33].
3. Integral-type BLF method [34].
4. Self-adaptive RBFNN method [35].
5. Finite-time PPC method [36].

In this simulation, to further evaluate the robustness of the controller, increasingly complex disturbances and actuator hysteresis were introduced to the system. The disturbance $d_{2}$ is characterized as a 2-s periodic square wave, which is a high-frequency disturbance. Moreover, $d_{4}$ represents unmodeled dynamics. They are modelled as

$$d_{2}\left(t\right)=\left\{\begin{array}{c} 0.3(0<t<1)\\ -0.3(1<t<2) \end{array}\right.,$$

$$d_{4}\left(t\right)=0.1\dot{x_{4}}-0.1{\ddot{x}}_{4}.$$

The actuator hysteresis is modeled as $\frac{d\kappa }{dt}=1.1\left|\frac{du}{dt}\right|+0.2\left(u-\kappa \right)$, where κ is the input with actuator hysteresis.

**Figure 10:**
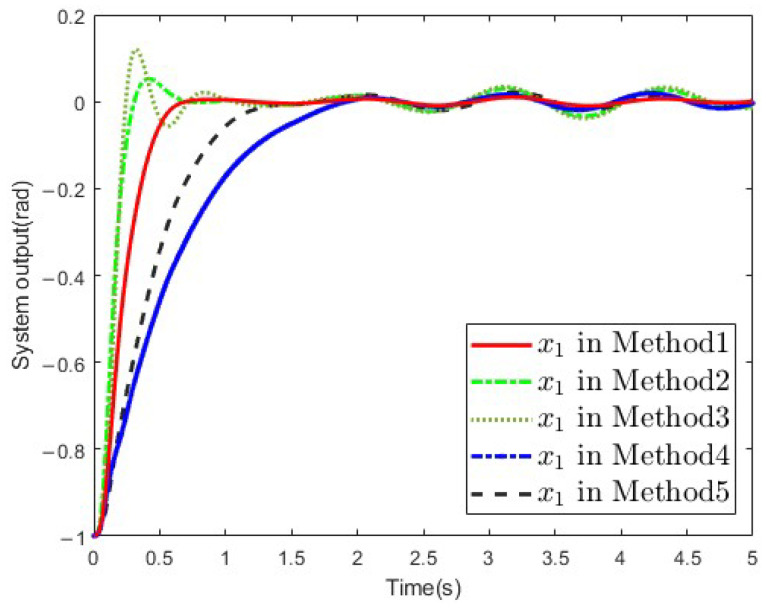
System output in different methods.

The Figure 10 presents the system output y in the five methods. As can be seen, the output of the SLFJM could be stabilized by the five methods, but the proposed method had faster convergence and smaller overshoot. The simulation results reflect the superiority of the proposed method.

## 5. Conclusions

For an SLFJM with sensor faults and external disturbances, a prescribed performance bounded-H_∞_ fault-tolerant controller without restrictions on the initial condition has been proposed. By employing an improved tangent-type BLF, the selection of the prescribed performance function is made completely independent of the initial state of the SLFJM position. An ICF was applied to ensure a smooth transition of the control signal from zero. Consequently, the workspace of the SLFJM controlled by the proposed controller is not constrained by the prescribed performance function. Compared to conventional BLF-based prescribed performance controllers, the proposed controller is applicable to a broader range of scenarios and offers enhanced safety. Finally, simulation results validate the effectiveness of the prescribed performance bounded-H_∞_ controller.

## Data Availability

No new data were created.

## Abbreviations

The following abbreviations are used in this manuscript:

SLMFJ	single-link flexible-joint manipulator
PPC	prescribed performance function
BLF	barrier Lyapunov function
ICF	input control function
IEF	initial expansion function
